# Association of DNA Methylation with Acute Mania and Inflammatory Markers

**DOI:** 10.1371/journal.pone.0132001

**Published:** 2015-07-06

**Authors:** Sarven Sabunciyan, Brion Maher, Sabine Bahn, Faith Dickerson, Robert H. Yolken

**Affiliations:** 1 Department of Pediatrics, Johns Hopkins University, Baltimore, MD, United States of America; 2 Department of Mental Health, Johns Hopkins School of Public Health, Baltimore, MD, United States of America; 3 Department of Chemical Engineering & Biotechnology, University of Cambridge, Cambridge, United Kingdom; 4 Sheppard Pratt Health System, Baltimore, MD, United States of America; UTHSCSH, UNITED STATES

## Abstract

In order to determine whether epigenetic changes specific to the manic mood state can be detected in peripheral blood samples we assayed DNA methylation levels genome-wide in serum samples obtained from 20 patients hospitalized for mania and 20 unaffected controls using the Illumina 450K methylation arrays. We identified a methylation locus in the *CYP11A1* gene, which is regulated by corticotropin, that is hypo-methylated in individuals hospitalized for mania compared with unaffected controls. DNA methylation levels at this locus appear to be state related as levels in follow-up samples collected from mania patients six months after hospitalization were similar to those observed in controls. In addition, we found that methylation levels at the *CYP11A1* locus were significantly correlated with three inflammatory markers in serum in acute mania cases but not in unaffected controls. We conclude that mania is associated with alterations in levels of DNA methylation and inflammatory markers. Since epigenetic markers are potentially malleable, a better understanding of the role of epigenetics may lead to new methods for the prevention and treatment of mood disorders.

## Introduction

Mood disorders are characterized by periods of mania and or depression. Studying these aberrant mood states at the molecular level has been challenging due to the inherent difficulties associated with obtaining samples from patients during acute mood episodes. Although the brain is the affected organ, postmortem samples represent a snapshot of the brain at death and are of limited use in examining specific mood states. However, the utility of peripheral samples, which are better suited for studying mood states, has been questioned [1,2].

The importance of the environment in the etiology of neuropsychiatric disorders is evident [3–6]. Many environmental factors, such as toxins and infectious agents, affect multiple organs and thus, are likely to alter biological processes in both the brain and peripheral tissues [7–9]. In addition, genetic and epigenetic lesions that are present in the germline or arise early in development will be present in multiple tissues. A recently published large study of DNA methylation in blood samples collected from schizophrenia patients and matched controls reports the existence of disease associated differences and convincingly demonstrates the suitability of peripheral samples in neuropsychiatric research [10]. The ability to assess the mental state of an individual concurrent with the collection of peripheral samples enables study of the natural history of psychiatric disorders over time. This approach potentially may lead to the discovery of diagnostic and prognostic biomarkers.

Accumulating evidence suggests that inflammation may be involved in the etiopathology of psychiatric disorders [11,12]. The aforementioned study reported that many of the differentially methylated regions in schizophrenia were related to markers of inflammation. A meta-analysis of cytokine measurements in bipolar disorder revealed that levels of tumor necrosis factor-α, soluble tumor necrosis factor receptor type 1 and soluble interleukin 2 receptor were elevated in manic patients [13]. Meta-analysis has also confirmed that C-reactive protein levels are elevated in bipolar disorder patients [14]. A recent analysis of psychiatric genome wide association studies discovered that disease associated polymorphisms are enriched in genes that function in immune signaling and histone methylation pathways [15]. This finding further supports the notion that the effects of inflammation may be mediated through epigenetic mechanisms and is consistent with the reported close association between DNA methylation and inflammation [16,17].

The purpose of the current study was to investigate whether DNA methylation differences may be associated with specific mood states. Large genetic studies have focused on collecting samples based solely on diagnosis and efforts to establish cohorts for specific mood states have been lacking. Based on current literature, the effect of DNA methylation on disease risk appears to be small and cohort sizes of at least several hundred may be necessary to detect the DNA methylation differences that are associated with specific mood states. Since we did not have access to an acute mania cohort that has sufficient statistical power, we were unable to conclusively test the hypothesis that DNA methylation at specific loci are altered in acute mania. Therefore, we opted to perform an exploratory study in 20 acute mania cases and 20 controls and used the obtained data for hypotheses generation. We picked the top hit from this analysis, the *CYP11A1* locus, and tested the hypothesis that DNA methylation levels associated with acute mania will change with disease trajectory. Thus, we assessed whether DNA methylation at this site changed between the time of hospitalization and six month follow up in a second cohort of acute mania patients. We also studied the association between DNA methylation at the *CYP11A1* locus and markers of inflammation in acute mania patients.

## Materials and Methods

### Samples

Blood samples were collected from consenting individuals admitted to Sheppard Pratt Hospital with acute mania. Inclusion criteria for the mania participants were: current admission to an inpatient or day hospital program for symptoms of mania or hypomania and potentially available for in-person follow-up six months later. Participants with mania could have an admission diagnosis of any one of the following: Bipolar I disorder, single manic episode; Bipolar I disorder, most recent episode manic; Bipolar I disorder, most recent episode mixed; Bipolar II disorder, most recent episode hypomanic; or Schizoaffective disorder, bipolar type (manic, hypomanic, or mixed state); absence of a primary diagnosis of alcohol or substance use disorder. The diagnosis of each psychiatric participant was established by consensus of the research team based on the SCID for DSM-IV Axis 1 Disorders—Patient Edition [18] and available medical records Psychiatric medication data were recorded from participant self-report and clinical charts and it was noted whether each patient was receiving each of the following types of medication at the time of the study visit: lithium, anti-convulsant mood stabilizer, second generation anti-psychotic, and/or antidepressant medications.

Blood samples were also obtained from individuals without a history of psychiatric disorder who were recruited from posted announcements at local health care facilities and universities in the same geographic area as the setting where the psychiatric participants were recruited. These control individuals were enrolled after they were screened to rule out the presence of a current or past psychiatric disorder with the Structured Clinical Interview for DSM-IV Axis I Disorders- Non-patient Edition [19]. Participants in both the mania and the control groups met the following additional criteria: age 18–65 (the control participants were aged 20–60); proficient in English; absence of any history of intravenous substance abuse; absence of mental retardation; absence of HIV infection; absence of serious medical disorder that would affect cognitive functioning; absence of a primary diagnosis of alcohol or substance use disorder.

The human studies were conducted in accordance with all institutional regulations and policies. The studies were reviewed and approved by the Sheppard Pratt Institutional Review Board. All participants provided written informed consent after the study procedures were explained. This consent procedure was approved by the IRB. The work described was carried out in accordance with The Code of Ethics of the World Medical Association (Declaration of Helsinki) for experiments involving humans.

Our study is comprised of serum samples collected from 34 individuals hospitalized with mania. A follow up sample was acquired from the same 34 individuals during a six-month follow up visit. Participants were classified as to whether they had been hospitalized for a new mood episode in the six month follow-up period. From the sample set of 34 individuals with mania, we selected 20 individuals (case group I) who were Caucasian and less than 50 years of age (see Table 1). These criteria enabled us to match the demographics of the control group as closely as possible and minimize the effect of age and race in our methylation studies. Samples collected from these individuals at the time of initial hospitalization and samples from 20 unaffected controls were used for the genome wide DNA methylation assays (Table 1). Bisulfite pyrosequencing was performed on these samples to validate the microarray results. DNA methylation at the *CYP11A1* locus was measured using pyrosequencing in samples from the remaining 14 individuals with mania (case group II) during hospitalization and at a six month follow up (Table 1). The original 20 samples were not included in this analysis because their DNA was depleted in the initial analyses.

**Table 1:** Cohort Demographics.

	Controls	Case Group I	Case Group II
Sex	6M/14F	8M/12F	3M/11F
Age	26.5(6.7)	36.6(6.6)	37.2(12.5)
Race	20 Caucasian	20 Caucasian	8 Caucasian, 6 non-Caucasian
RBANS (total)	86.85(9.5)	76.35(13.8)	74.93(12.5)
Disease Onset	NA	14.28(5.9)	18.03(7.7)
Disease Duration	NA	21.86(16.8)	18.16(13.6)
PANSS (total)	NA	72.45(12.1)	75.93(9.5)

The sex, mean age in years, race, Repeatable Battery for the Assessment of Neuropsychological Status (RBANS) score, mean disease onset in years, mean duration of disease in years and Positive and Negative Syndrome Scale (PANSS) total score are displayed for study participants. The standard deviation for each mean is shown in parenthesis.

Patients with autoimmune disorders and those who were using anti-inflammatories or immunomoduliatory drugs were not excluded from the study. In case group I, one individual had a diagnosis of diabetes mellitus and another was diagnosed with psoriasis. In case group II, 1 individual had a diagnosis of psoriasis, 1 had immune thrombocytopenic purpura, 1 had rheumatoid arthritis and 1 had multiple sclerosis. Note that samples from case group II were only used to determine DNA methylation levels at 6 month follow up and were not included in the regression analysis with inflammatory markers.

### Inflammatory Marker Measurements

A total of 159 inflammatory markers were measured by multi-analyte testing and solid phase enzyme immunoassay using previously described methods [20,21]. A full description of the multiplexed antigen immunoassay, including the list of serum proteins and small molecules assayed by this platform has been published [21].

### Whole Genome Methylation Arrays

Cellular material and clot was removed from whole blood using centrifugation. DNA was then extracted using the Qiagen RNeasy kit from serum samples collected from 20 controls and 20 patients admitted for acute mania and similar in the demographic variable of age, sex and race. Five hundred nanogram of DNA was then bisulfite converted and hybridized to Illumina 450K Methylation Arrays using the manufacturers recommended protocol at the Johns Hopkins CIDR core laboratory.

### Data Analysis of Methylation Arrays

The beta methylation values were obtained using the minfi bioconductor package in R. Wilcoxon Rank Sum test were used to identify differentially methylated loci. A *p<*0.05 was used as the threshold for statistical significance without adjusting for multiple comparisons to identify CpG loci for further analysis.

### Pyrosequencing

Three hundred nanograms of genomic DNA was bisulfite converted using the Zymo EZ Methylation Gold Kit and following the manufacturers recommended protocol. Nested PCR was performed on the bisulfite converted DNA using CpG unbiased primers. Primers TATTGGTAGTTTGGTAATTTTTTTT and AAAACCAACCCTAAATTTTCTTATTC were used for the original PCR reaction whereas primers TTGTTTTGTATGGATAGTGTTGGAG (5’ Biotinylated) and ATATTACATAAAACCCCCAAAAACC were used for the nested PCR reaction. The primer TACAATTAACAAATTCACTTTAA was used for sequencing.

### Regression Analysis

Linear regression analysis was performed between DNA methylation levels at the *CYP11A1* locus and inflammatory marker measurements. The DNA methylation levels as determined by pyrosequencing in 20 individuals hospitalized with mania (Case group I) and unaffected controls were used for the regression analysis. Regression analysis was performed separately for cases and controls. Bonferonni correction was used to correct for multiple testing.

## Results

**Fig 1:**
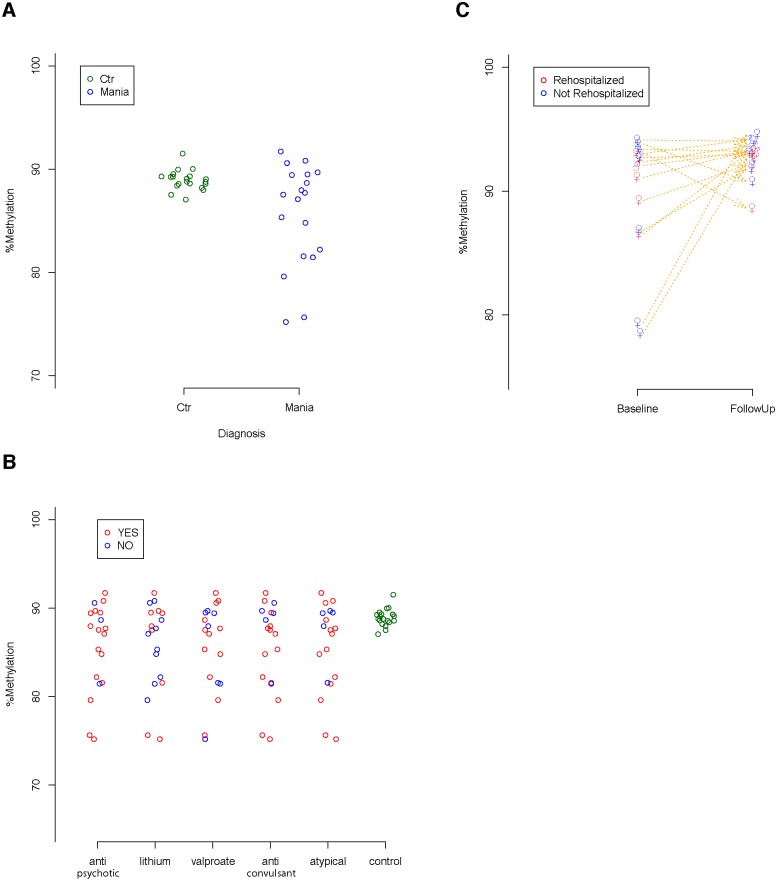
DNA methylation at the CYP11A1 methylation locus in acute mania. A) DNA methylation between cases and controls B) DNA methylation with anti-psychotic, lithium, valproate, anti-convulsant other than lithium, atypical anti-pscyhotic use and controls C) DNA methylation at baseline and at six month follow up.

We performed genome wide DNA methylation analysis in 20 individuals hospitalized with acute mania (case group I—see Table 1) and 20 unaffected controls. We found a single locus that differed between the individuals hospitalized with mania and controls (p<.006, Fig 1A). This pyrosequencing validated locus was a CpG site (chr15:74645873 genome build hg19) located near the cytochrome P450, family 11, subfamily A, polypeptide 1 (*CYP11A1*) gene,. The levels of methylation of this locus did not differ by covariates such as sex (*p* = 0.49 *t* = -0.705) and age (*p* = 0.48, *t* = 0.712) Within the group of individuals with mania we did not find an association between levels of methylation of this locus and class of medication received (Fig 1B).

To determine whether DNA methylation of *CYP11A1* is associated with the manic state, we performed bisulfite pyrosequencing on 14 additional mania patients (case group II—see Table 1) from whom samples were collected at the time of hospitalization and at a six month follow up. These additional samples are from individuals with mania recruited with the same selection criteria and methods as the previously described 20 samples from mania patients used for genome wide DNA methylation analysis. The comparison of DNA methylation levels between samples collected during hospitalization and samples collected from the same individuals six months later revealed a trend for increased DNA methylation in the follow up samples (*p* = 0.035, *t* = 1.9683) (Fig 1C). The DNA methylation levels in the follow up samples, which were similar to the levels measured in controls, did not correlate with sex of the patient or whether the patient had been re-hospitalized in the six month follow-up period. There were not sufficient numbers to evaluate the relationship between markers and clinical symptoms at follow-up.

**Fig 2:**
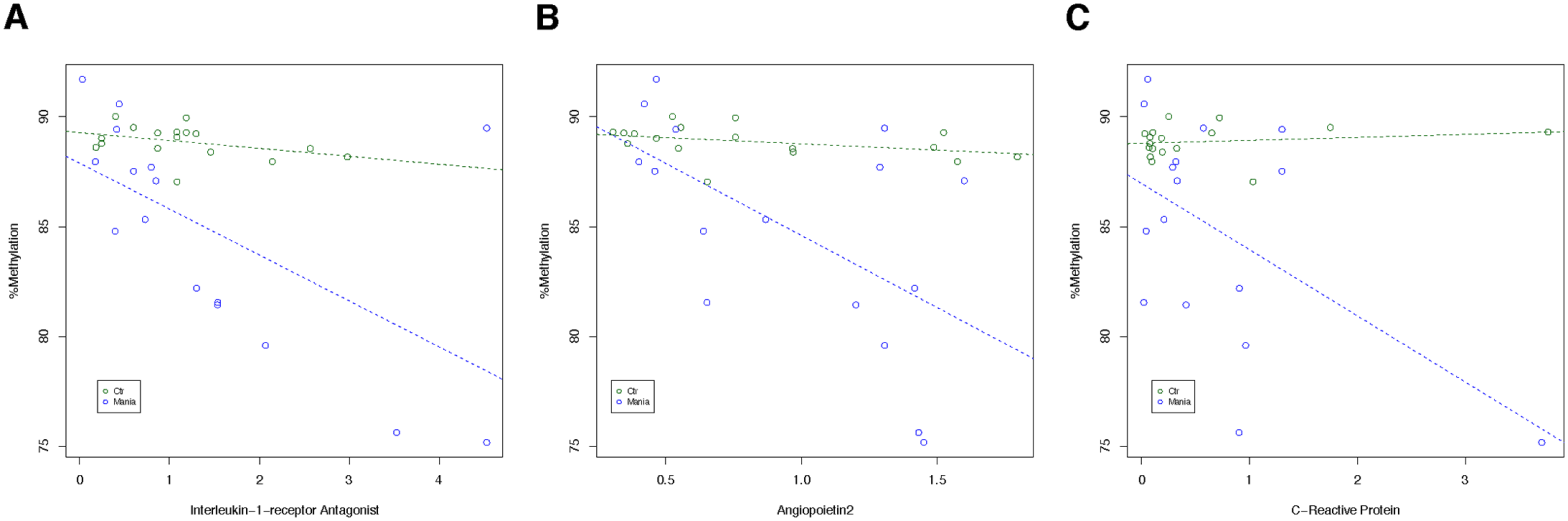
The correlation between DNA methylation at the CYP11A1 locus and inflammatory markers. Percent DNA methylation is plotted on the Y-axis against A) Interleukin-1 receptor antagonist B) Angiopoietin-2 and C) C-Reactive Protein levels on the X-axis. A separate regression line is drawn for acute mania cases and controls.

We next examined whether inflammatory markers which have been reported to be extensively increased in mania were significantly associated with DNA methylation at the *CYP11A1* locus. We also compared the effects of inflammation on DNA methylation marks between cases and controls. The regression analysis we performed revealed that the levels of Interleukin-1 receptor antagonist angiopoietin-2, and C reactive protein were significantly associated with DNA methylation in acute mania cases but not controls (*p* = 2.42e-05 padj = 0.004 *f* = 15.74; *p* = 7.90e-05 padj = 0.013 *f* = 14.17; *p* = 9.15e-05 padj = 0.0145 *f* = 11.45, respectively), (Fig 2) after correcting for multiple testing. All of the associations between inflammatory markers and DNA methylation in the unaffected controls failed to surpass the threshold for statistical significance.

## Discussion

In this study we found significant associations between acute mania, DNA methylation and inflammatory markers. The longitudinal study design we employed enabled us to identify DNA methylation changes in the *CYP11A1* locus in our cohort. Our data indicate that DNA methylation at this locus is dynamic and fluctuates between the time of acute mania and follow-up 6 months later. The *CYP11A1* gene encodes for an enzyme that is involved in the synthesis of steroid hormones and is regulated by corticotropin [22,23]. More importantly, the *CYP11A1* gene is involved in neurosteroid synthesis in the brain [24]. Neurosteroids regulate GABAergic transmission [25,26] and their levels are altered in individuals with a history of mania and bipolar disorder [27]. Psychiatric medications such as fluoxetine [28], lithium [29], clozapine [30] and olanzapine [31] have also been demonstrated to modulate neurosteroid levels. The DNA methylation changes we identified for the *CYP11A1* locus are significantly correlated with several inflammatory markers suggesting a potential molecular basis for the association between inflammation and mania. Although further experiments are needed to replicate our results, we are encouraged by the robust DNA methylation differences we observed between acute mania cases and unaffected controls.

Currently, cohorts for conducting epigenetic studies on specific mood states pale in size to cohorts collected for mood disorder genetics studies. An important motivation for conducting this study was to determine whether it is worthwhile to establish cohorts of specific mood states to study epigenetic marks. Therefore, we performed an exploratory study to determine whether DNA methylation mark levels may be associated with acute mania. Our exploratory genome-wide screening of 20 acute mania cases and 20 controls did not yield any differences that surpassed the threshold for statistical significance after correcting for multiple testing. Since exploratory studies are typically underpowered and are not intended for hypothesis testing, using nominal differences for hypothesis generation is an accepted approach [32]. We hypothesized that methylation marks that associate with mania should be restored to the levels observed in controls at 6 month followup samples. Thus, we used a separate cohort and investigated whether methylation at the *CYP11A1* locus would be restored to levels observed in controls by pyrosequencing. Our data suggest this to be the case, as the DNA methylation levels in the follow up samples were not statistically different from those observed in unaffected controls. The pliable nature of CpG methylation at the *CYP11A1* locus suggests that this site is associated with acute mania. However, more work is needed to determine whether methylation in this locus is involved in the etiopathology of mania. Regardless, the DNA methylation changes at the *CYP11A1* locus may have potential utility as a biomarker for predicting and or diagnosing individuals with mania. Replication studies and further research are necessary in order to determine the usefulness of the *CYP11A1* methylation locus as a biomarker.

The role of epigenetic marks in mood disorders is consistent with the cyclical nature of these illnesses. Static factors such as genotype cannot account for the episodic manic and depressive episodes that occur in mood disorders. Conversely, the plastic nature of epigenetic marks makes them ideal candidates for the molecular triggers that may induce aberrant mood states. This notion is supported by the fact that a commonly used mood stabilizer, sodium valproate, is a histone deacteylase inhibitor and is known to alter epigenetic marks [33]. An association between epigenetic marks and mental illness is also provided by recent studies that report a link between DNA methylation and inflammatory markers in schizophrenia samples [10]. In the current study, we provide further evidence to support this hypothesis by demonstrating that *CYP11A1* DNA methylation levels correlate with the acute manic state. The reasons for changes in time are not known with certainty but might be related to changes in medications, diet, or exposure to infectious agents. Our results suggest that DNA methylation markers change in individuals with acute mania and suggest that there is a difference in the response to environmental stimuli between mania cases and unaffected controls. Potentially, this differential response to environmental factors, which may act through epigenetic marks, may be a basis for disease. It is also possible that the abnormal response to inflammatory markers in patients may be associated with mood shifts observed in bipolar disorder and depression. However, our work cannot conclude that inflammation is associated with acute mania as our findings are limited to the periphery and we do not know the inflammatory state of the nervous system in these patients. Further work is warranted in order to delineate the association between epigenetic marks and environmental factors and identify the potential role of these interactions in disease pathology.

Although most methylation studies use cellular DNA, many DNA methylation biomarker studies in serum have been published [34–38]. These studies demonstrate that DNA methylation marks in serum may be associated with disease [34,35,37,38] and environmental exposures [36]. The presence in serum of low concentrations of DNA was assumed to originate from dying cells. However, this notion has been challenged by the discovery that extracellular vesicles, which are released from nearly all known cell types, contain genomic DNA [39–41]. The demonstration that extracellular genomic DNA can be transferred into distal cells and effect phenotype [42] suggests that circulating DNAs in serum may perform a function analogous to the signaling mediated by extracellular RNAs [43]. Thus, the use of serum samples for DNA methylation studies is appropriate and has the potential to yield biomarkers associated with disease. We also recognize that multi- analyte measurements yield different values for the same molecule compared to individual measurements. However, since we performed the analyte measurements using the same method for all samples, the case control differences we observe in DNA methylation response to inflammatory markers are likely to be real.

The value of peripheral samples in neuropsychiatric research may be limited by the fact that the brain is the affected organ and it is uncertain whether molecular changes associated with psychiatric disorder are reflected in the blood. However, the state of DNA methylation cannot be evaluated in the living brain and postmortem brain samples are limited in their utility since they only represent the state of the brain at death. Our findings in this study as well as other reports in the literature suggest that peripheral samples may be useful in terms of the evaluation of psychiatric disorders since they can represent the inflammatory state of the central nervous system [44]. Changes occurring during the course of neuropsychiatric disease can best be studied in peripheral samples. Longitudinal studies that collect samples in a prospective manner during the course of the illness are needed to develop prognostic and diagnostic biomarkers for disease. In addition, prospective, prenatal, pregnancy and twin studies can only be performed in peripheral tissues. Thus, the ability to ascertain disease related signatures in accessible samples is essential. Our work presents an initial attempt at uncovering disease state specific biomarkers using peripheral samples and demonstrates the feasibility of developing prognostic biomarkers for acute mania using this approach. Collection and molecular characterization of these types of samples is essential in the effort to develop biomarkers that are potentially predictive of detrimental disease states in bipolar disorder.
